# Occupational Medicine and Prevention of Chronic and Infectious Diseases

**DOI:** 10.3390/jcm12165298

**Published:** 2023-08-15

**Authors:** Giuseppe La Torre, David Shaholli, Corrado Colaprico, Maria Vittoria Manai, Salvatore Ammirati, Giorgia Mantione, Sabina Sernia

**Affiliations:** Department of Public Health and Infectious Diseases, Sapienza University of Roma, 00185 Rome, Italy; david.shaholli@uniroma1.it (D.S.); corrado.colaprico@uniroma1.it (C.C.); mariavittoria.manai@uniroma1.it (M.V.M.); salvatore.ammirati@uniroma1.it (S.A.); giorgia.mantione@uniroma1.it (G.M.); sabina.sernia@uniroma1.it (S.S.)

Occupational medicine is a clinical discipline that draws attention to the health of workers and their ability to work. Moreover, it concentrates on the physical, biological, chemical, and social context of the workplace. In this sense, occupational physicians can operate not only as specialist clinicians but also as all-round consultants, communicating with all levels of management on the relationship between work and health.

In recent years, the role and the interest of occupational medicine has changed, especially considering the changes in working conditions. It has evolved in a gradual and continuous way following challenges in response to new social, political, technological, and economic issues. Very recently, the main aim of occupational health has been deeply influenced by the globalization process of the world’s economies [1]. 

The new role of occupational medicine was experimented during the COVID-19 pandemic. Many occupational doctors were actively involved in organizing and delivering COVID-19 vaccines to workers, especially in the healthcare setting.

The main tool in the fight against COVID-19 has been the introduction of vaccines: the mRNA ones (Pfizer-BioNTech and Moderna) and the viral vector ones (AstraZeneca). New vaccines, classified as novel pharmaceuticals, are submitted to independent pharmacological-vigilance assessment through surveys dedicated to post-vaccination side effects. The study by Dziedzic et al. [2], conducted on 247 healthcare workers, including medical students and physicians, reveals moderate differences in the prevalence of local and systemic short-term adverse events in the sex and age groups after the first, second, and both doses of the vaccine, confirming their safety among HWs.

The COVID-19 pandemic made clear that there are vulnerable subgroups of Healthcare Workers (HCWs) for whom it is important to implement targeted prevention programmes focused on counselling and psychological assistance. Troisi and coll. [3] focused their attention to the relation between psychological well-being and occupational efficiency. The study aimed to predict levels of fear of COVID-19 in HCWs, using the “Big five Personality traits” and “Adult attachment style”. One hundred and one participants were recruited among HCWs of a COVID-19 hospital between June and August 2020. According to the study, older age predicted bigger fear of infection, and neuroticism and fearful attachment were independent predictors of fear of infection. These two personality traits could be considered more vulnerability traits due to their link with fear infection and the associated risk of a stress-related psychiatric condition.

The impact of COVID-19 on the mental health of HCWs has been thoroughly studied [4]. Sanchez et al. [5] focused their attention on the mediation of work engagement in the relationship between emotional intelligence (EI) and work performance in front-line HCWs throughout the COVID-19 pandemic. Data were collected from 1549 HCWs in Spanish hospitals. The results of this study revealed significant correlations between EI and all dimensions of work engagement (vigor, dedication, and absorption), as well as a positive correlation between EI and overall work performance. Furthermore, work engagement was found to be positively correlated with work performance, serving as a mediator between EI and job performance. Moreover, these findings suggest that HCWs with higher levels of EI tend to have superior work performance due to their enhanced work engagement as embracing emotional intelligence allows them to maintain focus and dedication amid uncertainty, positively impacting the quality of patient care. Overall, the study highlights the importance of fostering EI in managing the demands of a high-stress environment and emphasized the importance of work commitment in achieving optimal job performance in the healthcare sector.

Olaya and coll. [6] performed a systematic review and meta-analysis collecting data from cross-sectional studies reporting depression prevalence among HCWs from December 2019 to September 2020. Fifty-seven cross-sectional studies were considered in the meta-analysis, covering nurses, medical doctors, frontline HCWs, and other HCWs. Results have shown significant prevalence of depression among frontline workers (43%) compared to other HCWs categories (around 24–25% for each one). These findings emphasize the importance of addressing the mental health needs of HCWs through comprehensive support and interventions in order to provide appropriate psychological support and training to safeguard their wellbeing. Ongoing research and international collaboration are needed to understand and mitigate the impact of the pandemic on CWs mental wellbeing.

Studies conducted during the COVID-19 pandemic on HCWs revealed that the risk of contracting this infection disease was inversely related to the affective and cognitive aspects of individual wellbeing. However, the ability to tap into personal coping strategies linked to self-efficacy and meaning made it possible to adapt effectively to the situation in order to maintain an acceptable degree of wellbeing through processes of meaning-making and stress relief. In this regard, the study by Krok et al. [7] conducted on 225 healthcare workers confirmed that workers’ subjective wellbeing depends, to a large extent, on the risk level of contracting COVID-19 and motivational determinants, as well as the mediational factors carried out by stress and meaning-making.

The COVID-19 pandemic has resulted in a large quantity of deaths and health consequences on the general population. However, we cannot forget that occupational medicine is deeply involved in the prevention of other diseases, especially chronic ones. 

Various occupational risk factors, including work-related stress, prolonged work hours, or manual handling of heavy loads, are connected with increased cardiovascular risk. The study by Affinito and coll. [8] aimed to validate a new scale to determine and improve the risk of unfitness for work for cardiovascular risk determinants during health surveillance visits. The score, named “Cardiovascular Risk in Occupational Medicine” (CROM), was also confronted with the CVD risk scores already found in the literature. In this study, the link between the CVD risk factor and the workplace is confirmed, and a positive association between most of the cardiovascular risk determinants and the risk of unfitness for work is underlined. This article demonstrated the importance of accurate cardiovascular risk evaluation and adequate preventive approaches as part of occupational health surveillance and how the CROM score can be utilized to recognize workers who may benefit from targeted interventions to decrease their cardiovascular risk and prevent unsuitability for work.

Sometimes, occupational medicine focuses its attention on the rehabilitation process. In Sinkiewicz et al.’s study, titled ‘The effectiveness of Rehabilitation of Occupational Voice Disorders in a Health Resort Hospital Environment’ [9], they presented a rehabilitation programme (in a 24-day stay) of occupational voice disorders for 420 teachers (from 4 to 45 years of seniority) in order to evaluate its effectiveness.

Divided into three groups according to their seniority, they were assessed for jitter, shimmer, and NHR, before and after the intervention, in order to assess the maximum phonation time. They also underwent a perceptual assessment and a voice self-assessment using the GRBAS and VHI-30 scales, respectively. Both tests yielded positive results with a clear improvement in the parameters of jitter, shimmer, and NHR, and it was demonstrated that the early prevention of vocal disorders is important, as rehabilitation results are better in teachers with a shorter time of service.

The role of asbestos in the development of lung cancer and pleural mesothelioma has been clearly demonstrated, even if one fifth of mesothelioma is not explained by an exposure to asbestos. The study by Laurent et al. [10] was carried out on 2157 former workers previously professionally exposed to asbestos who underwent chest CT scans and pulmonary function tests in order to assess asbestos exposure and calculate a cumulative asbestos exposure index (CEI) for each subject. The aim was to evaluate the link between interstitial lung anomalies, asbestos exposure, and age in a population of retired workers previously occupationally exposed. The results showed that interstitial lung anomalies were present in 365 (16.7%) and emphysema in 444 (20.4%) participants, including a positive association with age but no association with CEI.

In modern and global occupational medicine, it is crucial to promote measures to improve the work environment that can combine adherence to legislations related to worker health and safety protection with health promotion programmes [11]. Workplace health promotion is one of the main factors to use in a cohort of HCWs with a minimum of one cardiovascular risk factor. A pilot study carried out by Rapisarda and coll. [12] involved 38 participants who were all from an emergency hospital. Patients were examined three times in a period of 12 months (T0; T6; T12), evaluating multiple clinical parameters like history of eating habits, objective examination, and BMI. According to the study results, by promoting healthier lifestyles and diets, a weight reduction and improvement in their lipid profile, blood pressure, work performance, and self-image perception are possible. There was also a considerable increase in the level of physical activity. 

A final consideration is given to one of the most widely used techniques for stress reduction. Chmielewski et al. [13] performed a study to assess whether mindfulness (i.e., a specific way of paying attention: conscious, non-judgmental, and present-moment oriented) can be associated with situational awareness (SA) levels between medical students involved in life-threatening scenarios during medical simulations. Using the Situation Awareness Global Assessment Technique at three levels, (1) data, (2) comprehension, and (3) projection, they found that nonreactivity, intended as minimal reactivity to stimulation, a better ability to focus, and an extended perspective compared to normal levels. In other terms, non-reactive attitudes toward internal and external experiences is really important, especially for the meticulous data collection of patients in life-threatening situations.
